# The Sleep and Diet of Elite Female Australian Rules Football Athletes Differ With Age and Season Phase

**DOI:** 10.1002/ejsc.70274

**Published:** 2026-10-21

**Authors:** Jackson Barnard, Monica Kelly, Michele Lastella, Spencer Roberts, Adrian Di Giovanni, Joshua Guy, Dominique Condo

**Affiliations:** ^1^ Centre for Sport Research, Institute of Physical Activity and Nutrition Deakin University Geelong Australia; ^2^ Appleton Institute for Behavioural Science Central Queensland University Wayville Australia; ^3^ School of Health Medical and Applied Sciences Central Queensland University Rockhampton Australia; ^4^ School of Health Medical and Applied Sciences Central Queensland University Cairns Australia

**Keywords:** athlete monitoring, recovery, sleep, sports nutrition

## Abstract

The Australian Football League Women's (AFLW) competition is comprised of a short, intensive competitive season, highlighting the importance of proper fuelling and recovery to support performance. Little is known about the dietary and sleep habits of AFLW athletes across a season, and the influence that age has on these behaviours. Therefore, this study aimed to determine the effects of season phase and age on dietary intake and sleep in elite AFLW athletes. Dietary intake and sleep were assessed in 22 AFLW athletes (25.9 ± 5.0 y) over 10 nights, including five consecutive nights during both pre‐season and in‐season. Dietary intake was quantified through self‐reported food diaries using a smartphone application (Easy Diet Diary), and sleep via a wearable monitoring device (Oura ring). Restricted maximum likelihood mixed models were used to determine variations in dietary intake and sleep in AFLW players between season phases (i.e., pre‐season vs. in‐season) and age. In‐season protein (115.15 ± 35.14 vs. 126.48 ± 40.10 g, *p* = 0.026) and fat intakes (72.93 ± 29.55 vs. 83.43 ± 33.14 g, *p* = 0.012) were significantly lower compared to pre‐season, with no differences in sleep measures observed between season phases. While age did not influence dietary intake, each additional year of age was associated with 8 min less sleep (*p* = 0.001) and 4 min earlier wake times (*p* = 0.032). These findings suggest that protein and fat intakes fluctuate across the seasons, with increasing age being a non‐modifiable risk factor for shorter sleep durations within AFLW athletes.

## Highlights

- Sleep measures remained unchanged between pre‐season and in‐season in AFLW players
- Increasing age was associated with shorter sleep duration and earlier wake times
- In‐season protein and fat intakes were lower than pre‐season intakes

## 1 Introduction

Australian Rules Football is a field‐based, physically demanding, and technical game (Clarke et al. 2018). The Australian Football League Women's (AFLW) competition is condensed into an 11‐week fixture, with this intense scheduling emphasising the importance of recovery practices that support readiness for subsequent training and competition. Sleep and nutrition are two key recovery behaviours that assist with muscle repair, glycogen repletion, immune function, and cognitive performance (Walsh et al. 2021; Kerksick et al. 2018). Though there are no specific sleep recommendations for athletes, it is believed that athletes require more than general recommendations of 7–9 hours to adequately recover from training and competition stressors, with athletes reporting a subjective sleep need greater than 8 hours to feel well‐rested (Sargent et al. 2021). However, previous data show that AFLW athletes achieve approximately 7.1 h on average in the preseason (Roberts et al. 2021; Lastella et al. 2021; Mascaro et al. 2024). Similarly, underconsumption of carbohydrates, iron, and calcium has been observed among AFLW athletes during the preseason compared with athlete‐based guidelines (S. L. Jenner et al. 2019), suggesting that recovery practices may not adequately support training and competition demands.

Most research investigating the sleep and dietary practices among AFLW athletes has been confined to the off‐season, limiting understanding of how these recovery behaviours may change across the competitive season. Currently, no studies have investigated changes in sleep across an AFLW season, while the literature within AFL men's remains equivocal, with some evidence suggesting there may be a reduction in sleep efficiency and wake after sleep onset (WASO) in the preseason compared to in‐season (Lalor et al. 2018). Given that training volume and scheduling vary across an AFLW season—factors known to influence sleep (Roberts et al. 2019)—understanding seasonal changes may help identify periods in which recovery is particularly vulnerable and inform targeted recovery strategies. Furthermore, there is a paucity of literature investigating seasonal changes in the dietary intake of AFLW players, with one study observing reductions in protein and increases in fat intake at the end of competition compared to the start of preseason (Otte et al. 2023). Similar recovery‐related challenges have also been observed in other female team‐sports athletes. For example, elite female basketball players have demonstrated impaired sleep following increased in‐season demands (Staunton et al. 2017), while female soccer players have reported greater energy, carbohydrate, protein, and fat intakes during the preseason compared to the latter stages of the season (Clark et al. 2003). Collectively, these findings suggest that sleep and dietary behaviours may fluctuate across a season in female team‐sport athletes, however, sport‐specific differences in training demands and competition scheduling highlight the need for AFLW‐specific investigation. Considering the critical role that protein and sleep play in supporting recovery, adaptation, and performance (Walsh et al. 2021; Jäger et al. 2017), greater understanding of these behaviours across the season may help inform athlete support strategies to optimise health and performance.

To date, no evidence has investigated the influence of age on the sleep and dietary habits of AFLW players. An AFLW playing list typically includes athletes ranging from 18 years to over 30 years of age, potentially resulting in differences in recovery and nutrition behaviours. While chronological age may be associated with factors such as accumulated playing experience and exposure to nutrition education (Walsh et al. 2021; Trakman et al. 2018), the influence of age on these behaviours in AFLW athletes remains unclear. Among sub‐elite and elite athletes from a range of sports, data suggest middle‐aged athletes (25–40 years) sleep less than their younger counterparts (18–24 years) (Vlahoyiannis et al. 2021), suggesting age‐related differences in sleep may exist in the AFLW. Similarly, age and playing experience have been seen to influence the dietary intake of AFL mens's (AFLM) players, with more experienced players reporting higher protein intakes than younger teammates (Bilsborough et al. 2016). Given the important role of proteins in recovery and adaptation (Jäger et al. 2017), such differences may have implications for athlete recovery. However, whether similar patterns exist in AFLW players remains unknown, as findings from AFL men's data may not be directly translatable due to differences in competition maturity, athlete pathways, and greater access to resources within the men's competition (Condo et al. 2019).

The aims of this prospective cohort study were to: (i) compare sleep outcomes and dietary intake between pre‐season and in‐season periods; (ii) compare dietary intake to athlete‐based guidelines; (iii) investigate the effect of age on sleep outcomes and dietary intake; and (iv) determine whether there is an interaction between season phase and age on the sleep outcomes and dietary intake of AFLW players.

## 2 Methods

### 2.1 Participants

Twenty‐two elite female AFLW players participated in this prospective cohort study (age, 25.9 ± 5.0 y; weight, 69.3 ± 6.9 kg; height, 172.4 ± 6.3 cm). All participants were voluntarily recruited from one club competing in the AFLW (Tier 3 athlete classification (McKay et al. 2021)), with participants required to be listed on the active player list for inclusion. This study was conducted in accordance with the Declaration of Helsinki, with ethical approval obtained from [CQUniversity Human Research Ethics Committee (approval number 0000022020)]. All participants provided written informed consent before participation.

#### 2.1.1 Experimental Design

A prospective observational study was conducted whereby habitual nutrition and sleep data were collected over five consecutive nights during the 2020 AFLW competitive season and pre‐season periods, respectively, with the same data collection timepoints achieved for all players. The pre‐season monitoring period was conducted during a typical training week, whereas the in‐season monitoring period included an afternoon match (1:10 p.m.) on the penultimate day of data collection.

#### 2.1.2 Dietary Intake

Habitual dietary intake was assessed via self‐reported food diaries using the Easy Diet Diary smartphone application (Xyris software, Canberra, Australia). As self‐reported dietary assessments are susceptible to underreporting and participant reactivity (Capling et al. 2017), players were instructed to record all food and drinks prospectively throughout each monitoring period. Photographs of meals were encouraged, but not mandatory, and were used to assist with the identification of inconsistencies within dietary records (Condo et al. 2021). An accredited sports dietitian reviewed all entries for completeness and consistency, conducting follow‐up where required, before dietary intake was quantified using the Australian‐specific dietary software program FoodWorks (version 9; Xyris software, Canberra, Australia). Dietary variables extracted from the data were energy (kJ), protein (g), carbohydrates (g), sugar (g), fat (g), saturated fat (g), sodium (mg), iron (mg), fibre (g) and calcium (mg) content of each meal and daily totals. Energy and macronutrient intakes (i.e., protein, carbohydrates, fat) were also calculated relative to body mass to allow for relative comparisons, with body mass collected at the start of both collection periods.

#### 2.1.3 Sleep

Sleep/wake behaviours of the players were measured using an Oura ring (Generation 2; Ōura Health Ltd, Oulu, Finland), a finger‐worn multisensory ring. The Oura ring quantifies sleep/wake status using accelerometry, gyroscope, photoplethysmography, and peripheral skin temperature from integrated temperature sensors (Mehrabadi et al. 2020). Previous validation against polysomnography has displayed close agreement for total sleep time (87.8% within ± 30 min), WASO (85.4% within ± 30 min), and sleep latency (mean difference −1.0 ± 15.9 min) (de Zambotti et al. 2019). The default Oura ring algorithm for the quantification of sleep was utilised without modification, with participants providing self‐reported bedtimes and wake times within the Oura application. As players slept within their habitual environments during data collection, they underwent an information session on how to correctly wear the Oura ring prior to any data collection. The following sleep variables were extracted for analysis:

- Bedtime (hh:mm): the self‐reported clock time at which a participant went to bed to attempt to sleep.
- Wake time (hh:mm): the self‐reported clock time at which a participant woke up and stopped attempting to sleep.
- Sleep onset latency (SOL; min): the period of time between bedtime and sleep onset.
- Total sleep time (TST; h): the total amount of sleep obtained during a sleep period.
- Sleep efficiency (SE; %): the percentage of time in bed that was spent asleep.
- Wake after sleep onset (WASO; min): the total amount of time spent awake during a sleep period.

### 2.2 Statistical Analysis

All statistical analyses were conducted within StataBE 18 (StataCorp LLC, TX, USA). Data cleaning was performed to identify missing and corrupt data, with restricted maximum likelihood mixed models used to analyse data. Individual observations (e.g., nightly sleep) were retained rather than averaged, with participant identification numbers included as a random effect to account for repeated measurements within participants. Age, season phase (in‐season, pre‐season), and their interaction were fitted as fixed effects within each model, determining their association with dietary intake (i.e., energy, protein, carbohydrates, sugar, fat, saturated fat, sodium, iron, fibre, calcium) and sleep outcomes (i.e., bedtime, wake time, SOL, TST, SE, WASO). Missing observations were accommodated within the mixed models, allowing all available data to contribute to the analysis without imputation. Statistically significant associations were set at *p* < 0.05, with data presented as mean ± SD unless otherwise stated.

## 3 Results

Twenty‐two elite female Australian footballers from one AFLW team completed the study. There was no participant dropout within the study, with 67% of sleep, and 80% of dietary intake data obtained.

### 3.1 Dietary Intake

Compared with the pre‐season, in‐season intakes of total protein (115.15 ± 35.14 g vs. 126.48 ± 40.10 g, *p* = 0.026), protein relative to body mass (1.67 ± 0.54 g·kg^−1^ vs. 1.81 ± 0.63 g·kg^−1^, *p* = 0.048), total fat (72.93 ± 29.55 g vs. 83.43 ± 33.14 g, *p* = 0.012), and fat relative to body mass (1.07 ± 0.46 g·kg^−1^ vs. 1.22 ± 0.51 g·kg^−1^, *p* = 0.013) were lower, while all other nutrient measures were not significantly different across season phases (*p* > 0.05; Table 1). Age had no effect on dietary intake, and no age‐by‐season phase interaction was detected for any nutrient measures (*p* > 0.05).

**TABLE 1:** Dietary intake across season phases.

Nutrient	Overall	Season phase				Age		Season phase × age (*p*‐value)
		Pre‐season	In‐season	Difference (95% CI)	*p*‐value	β‐coefficient (95% CI)	*p*‐value	
Energy (kJ)	8240.14 ± 2368.59	8410.82 ± 2398.30	8044.79 ± 2333.23	−387.44 (−949.78, 175.10)	0.177	−65.11 (−241.59, 111.38)	0.470	0.468
Energy (kJ·kg^−1^)	120.77 ± 39.17	123.11 ± 39.52	118.09 ± 38.84	−5.27 (−13.61, 3.06)	0.215	−1.72 (−4.82, 1.37)	0.274	0.671
Protein (g)	121.17 ± 38.17	126.48 ± 40.10*	115.15 ± 35.14*	−11.26 (−21.16, −1.36)	0.026	1.00 (−1.12, 3.12)	0.354	0.114
Protein (g·kg^−1^)	1.75 ± 0.59	1.81 ± 0.63*	1.67 ± 0.54*	−0.15 (−0.31, −0.01)	0.048	0.00 (−0.03, 0.04)	0.825	0.180
Carbohydrates (g)	182.87 ± 76.61	176.92 ± 73.94	189.69 ± 79.46	11.70 (−5.08, 28.48)	0.172	−4.65 (−10.67, 1.36)	0.129	0.394
Carbohydrates (g·kg^−1^)	2.70 ± 1.23^a^	2.61 ± 1.19	2.81 ± 1.28	0.18 (−0.07, 0.44)	0.157	−0.08 (−0.18, 0.02)	0.099	0.296
Sugar (g)	72.56 ± 33.65	70.58 ± 31.35	74.83 ± 36.16	1.68 (−6.13, 9.50)	0.673	−1.19 (−3.70, 1.32)	0.352	0.409
Sugar (g·kg^−1^)	1.07 ± 0.54	1.04 ± 0.50	1.11 ± 0.59	0.03 (−0.08, 0.15)	0.593	−0.02 (−0.06, 0.02)	0.313	0.556
Fat (g)	78.53 ± 31.87	83.43 ± 33.14*	72.93 ± 29.55*	−10.35 (−18.45, −2.26)	0.012	−0.07 (−2.13, 1.99)	0.947	0.246
Fat (g·kg)	1.15 ± 0.49	1.22 ± 0.51*	1.07 ± 0.46*	−0.15 (−0.27, −0.03)	0.013	−0.01 (−0.04, 0.03)	0.595	0.345
Saturated fat (g)	25.90 ± 12.05	26.73 ± 11.97	24.95 ± 12.14	−1.70 (−4.86, 1.46)	0.291	−0.18 (−0.95, 0.59)	0.648	0.425
Saturated fat (g·kg^−1^)	0.38 ± 0.19	0.39 ± 0.19	0.37 ± 0.19	−0.03 (−0.07, 0.02)	0.293	−0.01 (−0.02, 0.01)	0.420	0.505
Fibre (g)	25.43 ± 11.15	26.20 ± 10.59	24.56 ± 11.76	−2.08 (−4.89, 0.74)	0.148	−0.20 (−0.90, 0.49)	0.563	0.224
Calcium (mg)	928.84 ± 394.06^a^	965.96 ± 392.68	886.34 ± 393.71	−99.98 (−202.54, 2.58)	0.056	3.85 (−20.00, 27.71)	0.752	0.287
Sodium (mg)	2208.07 ± 1157.02	2174.42 ± 1033.56	2246.59 ± 1289.27	88.39 (−226.75, 403.54)	0.582	−56.88 (−115.84, 2.09)	0.059	0.309
Iron (mg)	11.32 ± 5.18^a^	11.45 ± 4.21	11.17 ± 6.12	−0.27 (−1.66, 1.12)	0.702	−0.18 (−0.46, 0.10)	0.205	0.194

*Note:* Values are presented as mean ± SD.

^a^ Intake below recommendations from sports nutrition guidelines (Kerksick et al. 2018; National Health and Medical Research Council 2006).

^*^ Statistical significance between season phase (*p* < 0.05).

### 3.2 Sleep

Across season phases, there were no significant differences in any sleep metrics (Table 2). For every 1‐year increase in age, there was an associated reduction in sleep duration and wake time by 8 min (*p* = 0.001) and 4 min (*p* = 0.032), respectively. No other sleep outcomes were affected by age, with no age‐by‐season phase interaction detected for any sleep metric (*p* > 0.05).

**TABLE 2:** Sleep measures across season phases.

Sleep metric	Overall	Season phase				Age		Season phase × age (*p*‐value)
		Pre‐season	In‐season	Difference (95% CI)	*p*‐value	β‐coefficient (95% CI)	*p*‐value	
Bedtime (hh:mm)	23:05 ± 01:16	23:04 ± 00:48	23:05 ± 01:38	00:02 (−00:16, 00:22)	0.780	00:02 (−00:01, 00:05)	0.179	0.810
Wake time (hh:mm)	07:40 ± 01:09	07:41 ± 01:07	07:39 ± 01:11	00:02 (−00:13, 00:18)	0.728	−00:04** (−00:08, −00:00)	0.032	0.130
Sleep onset latency (min)	11.56 ± 7.95	11.82 ± 8.13	11.26 ± 7.78	−0.61 (−2.73, 1.50)	0.570	−0.12 (−0.45, 0.20)	0.462	0.164
Total sleep time (h)	7.48 ± 1.18	7.36 ± 1.19	7.61 ± 1.15	0.27 (−0.02, 0.56)	0.071	−0.08** (−0.13, −0.03)	0.001	0.056
Sleep efficiency (%)	87.90 ± 4.46	87.52 ± 4.50	88.32 ± 4.40	0.67 (−0.42, 1.76)	0.230	0.05 (−0.20, 0.30)	0.711	0.759
Wake after sleep onset (min)	61.97 ± 25.34	63.40 ± 25.04	60.36 ± 25.70	−2.00 (−8.13, 4.14)	0.523	−1.01 (−2.41, 0.39)	0.156	0.273

*Note:* Values are presented as mean ± SD.

^**^ Statistical significance with age (*p* < 0.05).

## 4 Discussion

This study aimed to compare the sleep and dietary behaviours between the pre‐season and the competitive seasons of AFLW players, assess dietary intake relative to sports nutrition recommendations, and examine the influence of age and season phase on these behaviours. Key findings were that AFLW players had lower fat and protein intakes in‐season compared with pre‐season players, and that increasing age was associated with shorter sleep duration and earlier wake times. Although protein requirements were met throughout the season (1.8 ± 0.6 g·kg^−1^), average intakes of carbohydrates (2.7 ± 1.2 g·kg^−1^), calcium (928.8 ± 394.1 mg), and iron (11.3 ± 5.2 mg) were below current sports nutrition recommendations and nutrient reference values for female athletes (Kerksick et al. 2018; National Health and Medical Research Council 2006). Further, mean sleep durations met minimum guidelines for general populations (7.5 ± 1.2 h) (Hirshkowitz et al. 2015), however, players failed to reach 8 hours of sleep throughout each season phase (Sargent et al. 2021). In addition, wake after sleep onset exceeded 60 min across both season phases, suggesting that sleep continuity did not align with good sleep quality (Ohayon et al. 2017).

### 4.1 Dietary Intake

This was the first study to investigate the effect of season phase and age on the dietary intake of AFLW players. The evidence reported here of lower fat and protein intakes in‐season compared to pre‐season is consistent with previous findings in female footballers, where consumption of each macronutrient was higher during the pre‐season than in‐season (Otte et al. 2023; Clark et al. 2003). Typically, the pre‐season period involves higher training loads and manipulation of body composition due to the absence of match‐day performance pressures (Otte et al. 2023; S. Jenner et al. 2021), with higher protein intakes helping to maintain muscle mass and promote adaptation to training (Jäger et al. 2017). To further assess the periodisation of dietary intake in AFLW players, future research should measure macronutrient intake across more timepoints, allowing for trends in dietary intake to be analysed within season (i.e., multiple match days) and pre‐season periods (i.e., early and late pre‐season stages).

Dietary intake was not influenced by age in this study. This has been observed previously in AFLM athletes, with energy and carbohydrate intakes being similar between inexperienced (< 4 years) and experienced (≥ 4 years) players (Bilsborough et al. 2016). However, experienced AFLM players consumed more protein in‐season than their inexperienced counterparts (Bilsborough et al. 2016), whereas no age‐related differences in protein intake were observed in the present study. Furthermore, independent of age, overall energy and carbohydrate intakes were lower than recommendations (Thomas et al. 2016), which may be influenced by the semi‐professional nature of the AFLW competition, leading to a lack of nutrition knowledge, time, and finances to achieve optimal nutrition (Condo et al. 2019; Jagim et al. 2021). More qualitative research is required to understand the specific barriers to achieving optimal nutritional status in these semi‐professional athletes and identify any differences between experienced and inexperienced AFLW players.

On average, players met protein targets (1.4–2.0 g·kg^−1^ body mass) to support training adaptations and recovery (Jäger et al. 2017); however, similar to previous studies of AFLW athletes (S. L. Jenner et al. 2019; Otte et al. 2023; Condo et al. 2019, 2021), players in this study failed to meet carbohydrate, iron, and calcium recommendations irrespective of season phase. Achieving recommended dietary intakes for iron (18 mg·d^−1^), which is vital for oxygen transport, and calcium (1000 mg·d^−1^), critical for bone health, are crucial for the performance, longevity, and health of an athlete (National Health and Medical Research Council 2006; Beck et al. 2021). Female athletes are at an increased risk of iron deficiency due to increased iron losses through menstruation and exercise‐induced mechanisms (Suedekum and Dimeff 2005). When considered alongside the intake data observed in the present study, these findings support the consideration of routine iron status monitoring in AFLW players. Furthermore, AFLW training and competition involve moderate‐to‐high intensity exercise for 1–3 hours (Condo et al. 2019), with recommendations indicating that players should consume a carbohydrate intake of 5–12 g·kg^−1^ body mass to optimise fuelling and recovery (Thomas et al. 2016). In this study, however, the average carbohydrate intake was 2.7 g·kg^−1^ body mass, which falls below current sports nutrition recommendations and may not adequately support glycogen replenishment and recovery demands following training and competition (Thomas et al. 2016). However, it must be noted that these carbohydrate guidelines were primarily developed using data from endurance athlete‐based studies (Thomas et al. 2016), which may not take into account the unique physical and technical demands of Australian Rules Football (Clarke et al. 2018).

### 4.2 Sleep

Sleep measures remained unchanged across the season. This finding coincides with one AFL men's study, where there were minimal differences in sleep duration between season phases (Lalor et al. 2018). The mean sleep duration in the pre‐season of around 7 h in this study is consistent with previous AFLW sleep data collected during pre‐season periods (Roberts et al. 2021; Lastella et al. 2021). While unchanged, wake after sleep onset exceeded thresholds for good sleep quality (≥ 41 min) across both season phases (Ohayon et al. 2017). Given that sleep fragmentation may impair the restorative benefits of sleep independently of duration (Pilcher et al. 1997), these findings suggest that sleep continuity may be an important consideration when evaluating sleep behaviours in AFLW players. Given the semi‐professional nature of the AFLW competition, players often need to balance work and/or study alongside football. These additional commitments may necessitate consistency in the sleep/wake schedules and routines of players irrespective of season phase, potentially explaining this unchanged finding; however, this requires further investigation. Additionally, data collection did not coincide with a night‐game fixture—an in‐season factor known to negatively impact the sleep of athletes (Roberts et al. 2019). Overall, sleep duration remained below 8 hours across the season—the amount of sleep most athletes need to feel rested (Sargent et al. 2021). Athletes are exposed to many barriers to sleep throughout the season, with pre‐season factors such as increased training loads and in‐season factors including evening competition, travel, and pre‐game anxiety all potentially leading to sleep disruption (Roberts et al. 2019). To ameliorate inadequate sleep in AFLW players, further research should investigate barriers and enablers to sleep that these semi‐professional athletes experience to tailor sleep education and interventions.

Increasing age was associated with shorter sleep durations and earlier wake times among AFLW players. This coincides with previous data, whereby athletes aged 18–24 years on average sleep longer and wake later than their older counterparts (Randell et al. 2021). This age effect may be influenced by factors such as parenthood (Walsh et al. 2021), and older athletes typically being more morning‐orientated (Thun et al. 2015). In addition, with AFLW players working alongside their athletic careers, population‐based data suggest that individuals aged > 25 years are likely to work more hours than those aged < 25 years (Australian Bureau of Statistics 2022), indicating that employment may represent an additional sleep‐restricting barrier for older AFLW players. External employment commitments also force AFLW training sessions to be scheduled in the evening, with training generally undertaken between 6 and 8 p.m., thereby reducing sleep opportunity (Roberts et al. 2021). Given that these factors were not directly measured, future research should investigate whether contextual influences such as chronotype, employment commitments, training schedules, and competition demands contribute to the age‐related differences in sleep duration and wake time observed among AFLW players.

### 4.3 Strengths and Limitations

This was the first study to investigate the influence of season phase and age on the dietary intake and sleep of AFLW players. The use of self‐reported food diaries may have introduced underreporting and participant reactivity (Capling et al. 2017). Although the pre‐season and in‐season observations included in this study were collected prior to major COVID‐19‐related disruptions, the pandemic prevented the collection of a planned end‐of‐season timepoint, limiting the ability to characterise changes in sleep and dietary behaviours across the full AFLW season. Data were also collected for a total duration of 10 days (five in the pre‐season and five in‐season), which may not be representative of behaviours across the wider season. Furthermore, as this study was conducted within a single AFLW team, the modest sample size, while comparable with previous AFLW nutrition and sleep studies (Roberts et al. 2021; S. L. Jenner et al. 2019; Otte et al. 2023; Condo et al. 2019), may have limited the ability to detect small effects and reduced the generalisability of the findings across the wider AFLW competition. Despite these limitations, photo‐assisted recording and review by an accredited sports dietitian were used to improve the completeness and accuracy of dietary reporting, while inclusion of at least one weekend day provided a more representative assessment of habitual dietary intake. Food diaries were selected in place of weighed food diaries to reduce participant burden within this elite cohort.

## 5 Conclusion

We identified that AFLW players consume less fat and protein in‐season than during pre‐season, and that increasing age was associated with shorter sleep and earlier wake times. Though sleep was similar across the season, AFLW players typically sleep less than 8 hours throughout the entirety of the season. The average intake of carbohydrates, iron, and calcium did not align with sports nutrition guidelines, which may have implications for supporting optimal health, recovery, and performance in AFLW players. Future research should incorporate more frequent monitoring across season phases to better characterise changes in sleep and dietary behaviours, and identify periods during which recovery practices may be particularly vulnerable within an AFLW season.

## Funding

CQUniversity (seed funding).

## Conflicts of Interest

The authors declare no conflicts of interest.

## Data Availability

The data that support the findings of this study are available on request from the corresponding author. The data are not publicly available due to privacy or ethical restrictions.
